# Calcitonin attenuates cartilage degeneration and nociception in an experimental rat model of osteoarthritis: role of TGF-β in chondrocytes

**DOI:** 10.1038/srep28862

**Published:** 2016-06-27

**Authors:** Zhi-Hong Wen, Chi-Chieh Tang, Yi-Chen Chang, Shi-Ying Huang, Yen-You Lin, Shih-Peng Hsieh, Hsin-Pai Lee, Sung-Chun Lin, Wu-Fu Chen, Yen-Hsuan Jean

**Affiliations:** 1 Department of Marine Biotechnology & Resources, and Center for Translational Biopharmaceuticals, National Sun Yat-Sen University, Kaohsiung, Taiwan; 2 Department of Early Childhood Education, National Pintung University, Taiwan; 3 Section of Pathology, Pingtung Christian Hospital, Pingtung, Taiwan; 4 Department of Orthopedic Surgery, Pingtung Christian Hospital, Pingtung, Taiwan; 5 Department of Neurosurgery, Chang Gung Memorial Hospital-Kaohsiung Medical Center, Chang Gung University College of Medicine, Taiwan

## Abstract

We investigated the role of the calcitonin (Miacalcin) in the progression of osteoarthritis (OA) and in nociceptive behavior in an experimental rat model of OA and osteoporosis. OA was induced by anterior cruciate ligament transection (ACLT) of the right knee and by bilateral ovariectomy (OVX) in Wistar rats. Nociceptive behaviors (secondary mechanical allodynia and weight-bearing distribution of the hind paws) were analyzed prior to surgery and every week, beginning at 12 weeks after surgery, up to 20 weeks. At 20 weeks, histopathological studies were performed on the cartilage of the knee joints. Immunohistochemical analysis was performed to examine the effect of calcitonin on transforming growth factor (TGF)-β1 expression in articular cartilage chondrocytes. Rats subjected to ACLT + OVX surgery showed obvious OA changes in the joints. Animals subjected to ACLT + OVX and treated with calcitonin showed significantly less cartilage degeneration and improved nociceptive tests compared with animals subjected to ACLT + OVX surgeries alone. Moreover, calcitonin increased TGF-β1 expression in chondrocytes in ACLT + OVX-affected cartilage. **S**ubcutaneous injection of calcitonin (1) attenuated the development of OA, (2) concomitantly reduced nociception, and (3) modulated chondrocyte metabolism, possibly by increasing cellular TGF-β1 expression.

Osteoarthritis (OA), is a complex disease characterized by bone remodeling, synovium inflammation, and cartilage loss. Although OA is classically defined as a progressive degenerative disease of the articular cartilage, inflammation plays a key role in its pathogenesis^1^. The pain associated with OA is primarily localized to the affected joint, but a number of OA patients also exhibit increased nociception in adjacent or even remote areas of the body^2^. Patients with ruptured anterior cruciate ligaments (ACL) develop post-traumatic OA of the knee^3^.

Although ovariectomy (OVX) is a classical technique to induce osteoporosis, it also successfully induces OA^4^. Studies using animal models have shown that OVX can successfully induce OA in the rat articular cartilage^5^. The pathological changes observed in OVX rats were similar in nature to those observed very early in human OA, such as mild erosion and loss of proteoglycans, as described previously^6^.

Calcitonin is a 32-amino acid hormone produced by thyroid gland parafollicular cells that, similar to 1,25-dihydroxyvitamin D, increases calcium and phosphate uptake to counteract the effects of parathyroid hormone^7^. Calcitonin is approved for the treatment of postmenopausal osteoporosis, malignancy-associated hypercalcemia, and Paget disease, all of which involve accelerated bone turnover^8^. Clinical trials involving patients with spinal fractures and knee OA suggest that calcitonin has anti-nociceptive effects, as evidenced by the reduced consumption of analgesic drugs^9^. Bagger *et al*. demonstrated significant inhibition of not only cartilage degradation but also bone resorption with an oral formulation of calcitonin in a 3-month intervention study of healthy elderly women^10^. In cultured isolated chondrocytes and *in vitro* explants, calcitonin may stimulate collagen type II and proteoglycan synthesis^11^, suggesting potential anabolic effects of the hormone on cartilage. Although several positive effects of calcitonin on chondrocytes have been demonstrated, the biological mechanism underlying its potential direct effects on nociception and OA development remain unclear.

Transforming growth factor-beta (TGF-β) is a multi-functional cytokine involved in crucial biological processes such as extracellular matrix synthesis, cell proliferation and differentiation, and tissue repair^12^. Intra-articular injection of TGF-β induces an increase in proteoglycan synthesis and articular cartilage content in naïve murine knee joints^13^. Loss of TGF-β signaling in cartilage induces chondrocyte hypertrophy, leading to cartilage degeneration^14^, and pharmacological activation of the TGF-β pathway has therefore been proposed to preserve articular cartilage integrity during OA^15^. The aim of the present study was to assess the effect of calcitonin on OA development in ACL transection (ACLT)- and OVX-induced OA rats and the anti-nociceptive effect of calcitonin in OA rats by measuring TGF-β1 expression in the articular cartilage by immunohistochemistry.

## Methods

### Animals

The use of rats conformed to the Guiding Principles in the Care and Use of Animals approved by the Council of the American Physiology Society and was approved by the National Sun Yat-Sen University Animal Care and Use Committee (approval number PTCH-2-300-005-2). Three-month-old male Wistar rats (body weight = 295–320 g) were maintained under climate-controlled conditions on a 12-h light-dark cycle at 22–24 °C with a relative humidity of 50–55%.

### Surgical technique for induction of OA and Osteoporosis

OA was induced in rats by ACLT of the right knee; the left knee was not treated. Rats were anesthetized with 3% isoflurane in an oxygen/room air mixture (1:1). The surgical procedure was modified from the protocol described in previous studies^16^. Osteoporosis was induced in rats by bilateral OVX. The surgical procedure was modified from the protocol described in a previous study^6^. The animals were not immobilized after surgery and were allowed daily unrestricted cage activity. They were closely monitored for infections and other complications for 20 weeks after surgery.

### Experimental design and calcitonin treatment

The animals were divided randomly into 5 groups. Group I (naïve group; n = 8) rats did not undergo surgery or treatment. Group II (calcitonin 15 U group; n = 6) animals underwent no surgery and received 15 U calcitonin (Miacalcin; calcitonin salmon synthetic, Novartis, Basel, Switzerland) via subcutaneous injection. In group III (ACLT + OVX group; n = 8), rats underwent both ACLT and OVX surgeries, and received 0.1 mL distilled water via subcutaneous injection. Group IV was referred to as the ACLT + OVX + 3 U calcitonin group (n = 6) and the rats underwent both ACLT and OVX surgeries and received 3 U calcitonin via subcutaneous injection two times per week for 9 consecutive weeks, beginning 12 weeks after surgery until 20 weeks. Group V was referred to as ACLT + OVX + 15 U calcitonin group (n = 7) and the rats received the same treatment as group IV except the calcitonin dose was 15 U. Measurements of secondary mechanical allodynia and hind paw weight distribution were performed as nociceptive behavioral tests prior to and 12 through 20 weeks after surgery. Changes in knee joint width were measured on the same schedule. At 20 weeks after surgery, gross morphology and histopathological examinations were performed on the cartilage of the femoral condyle and tibial plateau of the knee joint. Immunohistochemical analysis was performed to examine the effect of calcitonin on TGF-β1 expression in articular cartilage chondrocytes.

### Assessment of nociception

#### Secondary mechanical allodynia

Allodynia was assessed with von Frey Filaments (North Coast Medical, Inc. Morgan Hill, CA, USA). The filament diameters corresponded to a logarithmic scale of the force exerted, and thus the perceived intensity was measured on linear and interval scales. The withdrawal threshold was determined by Chaplan’s up-down method involving the use of alternate large and small fibers to determine the 50% withdrawal threshold^17^. Each von Frey filament was applied to the plantar surface of the paw for 5 s. Briefly, when the rat lifted its paw in response to pressure, the filament size was recorded, and a weaker filament was then tested. The von Frey filament was applied to each paw for 5 trials at approximately 3-min intervals.

#### Weight-bearing distribution test

The effect of joint damage on weight distribution through the right (OA) and left (contralateral) knees was measured using a dual channel weight averager (Singa Technology Corporation, Taipei, Taiwan), which independently measures weight bearing by each hind paw. The change in hind paw weight distribution was used as an index of joint discomfort and was determined as described previously^18^,^19^. Briefly, rats were placed in an angled plexiglass chamber positioned so that each hind paw rested on a separate force plate. The force exerted by each hind limb (measured in grams) was averaged over a 5-s period. Each data point is the mean of three 5-s readings. The hind paw weight distribution is expressed as the difference in weight bearing between the contralateral and ipsilateral limbs.

#### Knee joint width, gross morphology, and histopathological findings of joints

Changes in knee joint width were measured bilaterally using calipers (AA847R, Aesculap, AG, Tuttlingen, Germany) prior to surgery and every week, beginning at 12 weeks after surgery, up to 20 weeks. The width of the knee joint was measured from the medial to the lateral aspect of the knee joint (at approximately the level of the medial and lateral joint lines). Immediately after sacrifice, each knee was examined for gross morphological changes, such as cartilage lesions, of the femoral condyle and tibial plateau as previously described^20^. Briefly, cartilage erosion was graded on a scale of 0–4, with 0 = normal surface appearance; 1 = minimal fibrillation or a slight yellowish discoloration of the surface; 2 = erosion extending to the superficial or middle layers; 3 = erosion extending to the deep layer; and 4 = erosion extending to the subchondral bone. The joints were sectioned 0.5 cm above and below the joint line, fixed in 10% neutral-buffered formalin for 3 days, and then decalcified for 2 weeks in buffered 12.5% ethylenediaminetetraacetic acid and formalin solution. The joints were then sectioned mid-sagittally, washed with tap water, placed in embedding cassettes for dehydration, clearing, and infiltration by an automatic tissue processor (Tissue-Tek, Sakura Finetek Japan Co., Ltd., Japan), embedded in paraffin blocks using a tissue embedding center (EC780-1; EC780-2, USA), and cut into 2-μm sections using a rotary microtome (HM340E, Microm, Biotechnical Services, Inc., San Diego, CA, USA) from the central weight-bearing surfaces of the femoral condyles and tibial plateaus of both knees. The sections were stained with hematoxylin and eosin and Safranin-O/fast green to assess the general morphology and matrix proteoglycan of the cartilage. Articular cartilage was graded under microscopic examination according to the Osteoarthritis Research Society International (OARSI) grading system^21^. This system comprises 6 histological grades and 4 histological stages. The total score (score = grade × stage) ranges from 1 point (normal articular cartilage) to 24 points (no repair).

#### Immunohistochemical staining for TGF-β1

Cartilage specimens were processed for immunohistochemical analysis as previously described^22^,^23^. Briefly, sections (2 μm) of the paraffin-embedded specimens were placed on slides, deparaffinized with xylene, and dehydrated in a graded series of ethanol solutions, and then endogenous peroxidase activity was quenched by incubation in 0.3% hydrogen peroxide for 30 min. The antigen was retrieved by enzymatic digestion with proteinase K (20 mM; Sigma, St. Louis, MO, USA) in phosphate-buffered saline (PBS) for 20 min. After 2 10-min washes with PBS, the sections were incubated with PBS containing 5% normal goat serum for 30 min to block non-specific binding. The slides were incubated with primary antibodies against anti-TGF-β1 (1:200 dilution, cat. ab92486; Abcam, Cambridge, UK; polyclonal rabbit antibody). The sections were then incubated for 90 min with biotinylated anti-rabbit IgG (Vector Labs, Burlingame, CA, USA) diluted 1:200 with 1% BSA in PBS. Thereafter, the sections were treated with the avidin-biotin complex technique using an ABC kit (Vectastain ABC kit; Vector Labs), followed by 3,3′-diaminobenzidine tetrahydrochloride (Vectastain ABC kit; Vector Laboratories) for 5 min. The different antigens present in each cartilage specimen were quantified by determining the number of positively stained chondrocytes in the entire thickness of cartilage, as previously described^23^. The cartilage was divided into 6 microscopic fields (3 each in the superficial and deep zones) (magnification, 400×), and the results were averaged. Prior to evaluating each OA specimen, an intact cartilage surface was identified for use as a marker to validate the results of morphometric analysis. The final results were expressed as the percentage of chondrocytes stained positive for each antigen (cell score), with a maximum score of 100% for each cartilage specimen. Each slide was reviewed by 2 independent readers who were blinded to the treatment groups.

In contrast, for double-immunofluorescent staining of TGF-β1 and articular chondrocyte markers (doublecortin)^24^, the cartilage sections were incubated with a mixture of anti-TGF-β1 (1:200 dilution, cat. ab64715; Abcam; monoclonal mouse antibody) and anti-doublecortin (ab18723; Abcam; polyclonal rabbit antibody) antibodies overnight at 4 °C, and then followed by a mixture of Alexa Fluor 488-conjugated chicken anti-mouse IgG antibody (1:400 dilution; cat. A-21200; Molecular Probes, Eugene, OR, USA; green fluorescence) and DyLight 549-conjugated donkey anti-rabbit IgG antibody (1:400 dilution, cat. 711-506-152; Jackson ImmunoResearch Laboratories Inc., West Grove, PA, USA; red fluorescence) for 40 min at room temperature. We then acquired the double-immunostaining images using Leica TCS SP5 II confocal microscope (Leica Instruments).

#### Data and statistical analysis

All continuous data were presented as the mean ± standard error of the mean (SEM). One-way analysis of variance (ANOVA) was used to test for significant differences among the means of various scores of experimental groups. To compare the mean differences between the treatment and naïve groups, the Student-Newman-Keuls method *post-hoc* test was used. The trends for the changes in nociceptive behavior and knee joint width were tested using repeated-measures ANOVA. Differences resulting in *P*-values of less than 0.05 were considered significant.

## Results

All rats in our study survived to sacrifice without infection.

### Nociceptive behavior (secondary mechanical allodynia and weight-bearing distribution)

**Figure 1:**
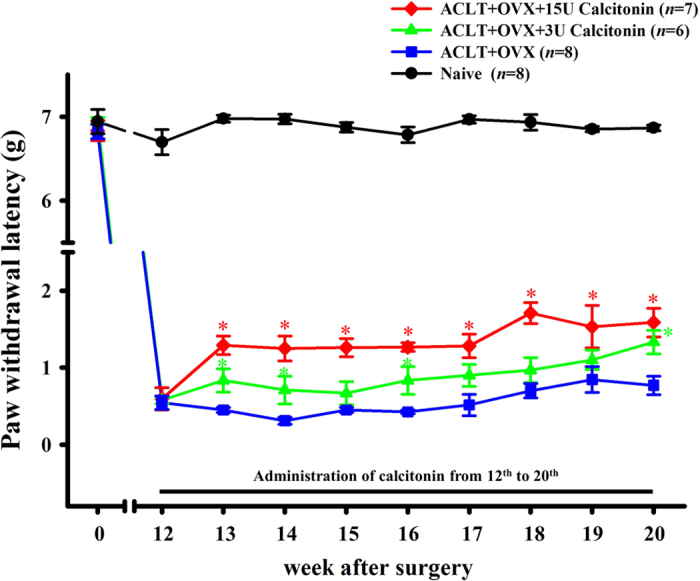
Time course of the anti-allodynic effect of calcitonin in ACLT-induced OA/OVX-induced osteoporosis nociception pain model. Data are the mean ± SEM of the ipsilateral hind paw of each group. *P < 0.05 vs the ACLT + OVX group.

Figure 1 shows the effects of calcitonin on secondary mechanical allodynia. The paw withdrawal latency was significantly increased in the ACLT + OVX + 3 U calcitonin group compared to the ACLT + OVX group at 13, 14, 16, and 24 weeks after surgery (0.83 ± 0.15 vs. 0.45 ± 0.03 g; 0.71 ± 0.18 vs 0.31 ± 0.04 g; 0.83 ± 0.18 vs 0.43 ± 0.03 g and 1.33 ± 0.15 vs 0.77 ± 0.12 g respectively; *P* < 0.05; Fig. 1). This effect was the same as that observed in the ACLT + OVX + 15 U calcitonin group than in the ACLT + OVX group at 13–20 weeks after surgery (1.29 ± 0.12 vs 0.45 ± 0.03 g; 1.25 ± 0.16 vs 0.31 ± 0.04 g; 1.26 ± 0.12 vs 0.45 ± 0.03 g; 1.27 ± 0.06 vs 0.43 ± 0.03 g; 1.28 ± 0.15 vs 0.52 ± 0.14 g; 1.71 ± 0.14 vs 0.7 ± 0.09 g; 1.53 ± 0.28 vs 0.85 ± 0.17 g and 1.59 ± 0.19 vs 0.77 ± 0.12 g, respectively; *P* < 0.05; Fig. 1).

**Figure 2:**
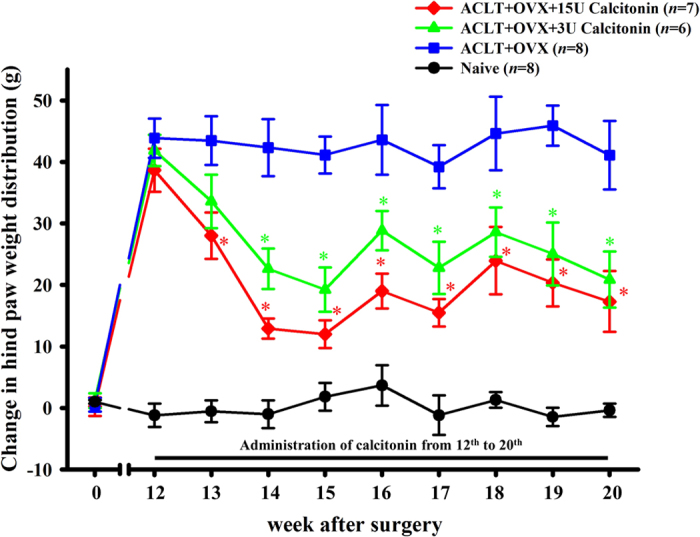
Time course of the effect of calcitonin on weight bearing of the contralateral (left) and ipsilateral (right) ACLT hind paws for 20 weeks after ACLT/OVX surgery. The baseline was set as the weight-bearing differential prior to surgery. Data are the mean ± SEM of each group. *P < 0.05 vs the ACLT + OVX group.

The weight-distribution differential between the OA and contralateral hind paws in the ACLT + OVX + 3 U calcitonin group was lower than that of the ACLT + OVX group at 14–20 weeks after surgery (22.62 ± 3.28 g vs 42.33 ± 4.64 g; 19.22 ± 3.60 g vs. 41.11 ± 3.01 g; 28.33 ± 3.18 g vs 4.3.59 ± 5.64 g; 22.77 ± 4.26 g vs 39.2 ± 3.51 g; 28.57 ± 4.00 g vs 44.61 ± 5.98 g; 25.03 ± 5.13 g vs 45.89 ± 3.27 g and 20.85 ± 4.57 g vs 41.09 ± 5.58 g respectively; *P* < 0.05; Fig. 2). The same trend was observed in the comparison between the ACLT + OVX + 15 U calcitonin and ACLT + OVX groups at 13–20 weeks after surgery (28.00 ± 3.75 g vs 43.46 ± 3.97 g; 12.91 ± 1.64 g vs 42.33 ± 4.64 g; 12.00 ± 2.27 g vs 41.11 ± 3.01 g; 19.00 ± 2.83 g vs 43.59 ± 5.64 g; 15.47 ± 2.24 g vs. 39.20 ± 3.51 g; 23.93 ± 5.47 g vs. 44.61 ± 5.98 g; 20.33 ± 3.81 g vs. 45.89 ± 3.27 g and 17.30 ± 4.95 g vs. 41.09 ± 5.58 g respectively; *P* < 0.05; Fig. 2). Moreover, no significant difference in nociception in the contralateral limbs was observed among the experimental groups (data not shown).

### Change in knee joint width

**Figure 3:**
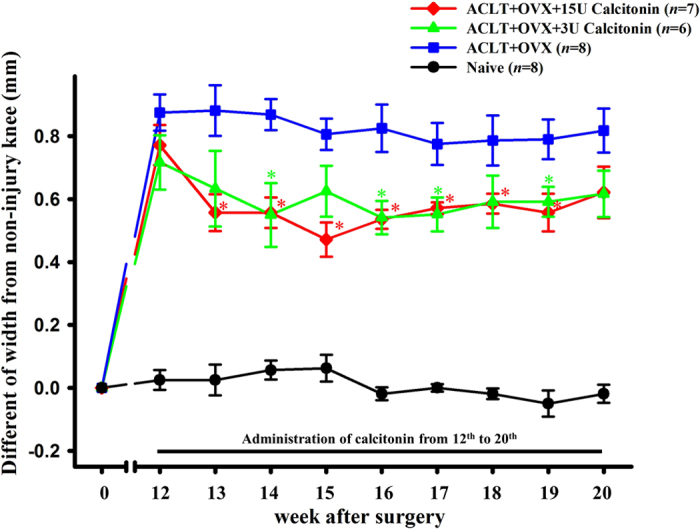
Time course of the effects of calcitonin on bilateral hind knee joint widths after ACLT/OVX. The data are the mean ± SEM of knee width, with baseline set as the widths prior to surgery. *P < 0.05 vs the ACLT + OVX group.

There was a significant decrease in the width of the hind limb knee joint in the ACLT + OVX + 3 U calcitonin group compared with the ACLT + OVX group at 14–20 weeks after surgery (0.55 ± 0.1 vs 0.87 ± 0.05 mm; 0.63 ± 0.08 vs 0.81 ± 0.05 mm; 0.54 ± 0.05 vs 0.83 ± 0.08 mm; 0.55 ± 0.05 vs 0.78 ± 0.07 mm; 0.59 ± 0.08 vs 0.79 ± 0.08 mm; 0.59 ± 0.05 vs 0.79 ± 0.06 mm and 0.62 ± 0.07 vs 0.82 ± 0.07 mm, respectively; *P* < 0.05; Fig. 3). The same trend was observed for the comparison between the ACLT + OVX + 15 U calcitonin and the ACLT + OVX group at 13–20 weeks after surgery (0.56 ± 0.06 vs 0.88 ± 0.08 mm; 0.56 ± 0.05 vs 0.87 ± 0.05 mm; 0.47 ± 0.05 vs 0.81 ± 0.05 mm; 0.54 ± 0.03 vs 0.83 ± 0.08 mm; 0.57 ± 0.02 vs 0.78 ± 0.07 mm; 0.59 ± 0.03 vs 0.79 ± 0.08 mm; 0.56 ± 0.06 vs 0.79 ± 0.06 mm and 0.62 ± 0.08 vs 0.82 ± 0.07 mm respectively; *P* < 0.05; Fig. 3).

### Gross morphology

Gross characteristics of cartilage degeneration, such as fibrillation, erosion and ulcer formation, and osteophyte formation, were observed in the femoral condyles and tibial plateau in the ACLT + OVX groups. Markedly less severe cartilage damage was observed in both groups treated with calcitonin. In the naïve and 15 U calcitonin groups, the cartilage was macroscopically normal, with a glistening, smooth surface, and no cartilage defects or osteophytes were observed. A significant difference (*P* < 0.05) in the gross morphology score was observed between the ACLT + OVX and the ACLT + OVX + 3 U calcitonin and ACLT + OVX + 15 U calcitonin groups (Table 1). The grades of cartilage damage in both calcitonin-treated groups were significantly lower than in the ACLT + OVX groups (Table 1). Naïve rats treated with 15 U calcitonin were normal in gross appearance (data not shown).

### Microscopic findings

**Figure 4:**
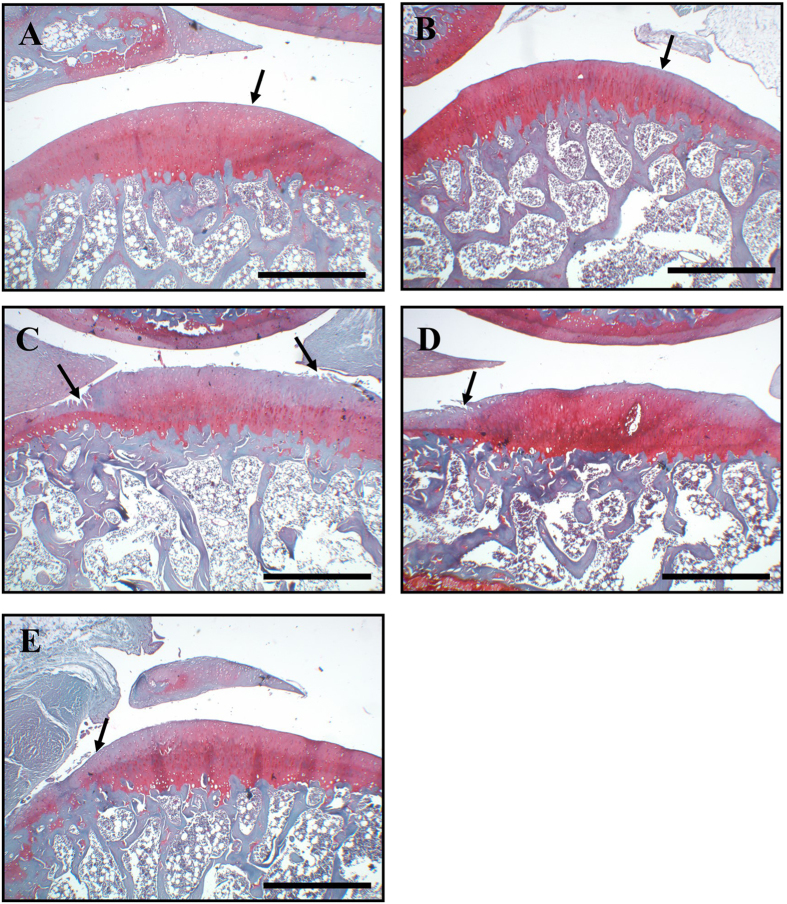
Histopathological evaluation of articular cartilage of the knee joint. In both the naïve (**A**) and calcitonin 15 U (**B**) groups, the surface of the superficial cartilaginous layer was smooth (arrow) and the cartilage matrix was prominently stained with H/E. Specimens from the ACLT + OVX group (**C**) showed decreased cartilage thickness, disappearance of the surface layer cells (arrow), a fissure extending into the transitional and radial zones, and chondrocyte hypocellularity in the transitional and radial zones. The specimens from the ACLT + OVX + 3 U calcitonin group (**D**) and ACLT + OVX + 15 U calcitonin groups (**E**) showed mild irregularity of the surface layer, fibrillation of and fissures within the superficial cartilaginous layer (arrow), and slight diffuse hypercellularity in the transitional and radial zones. Stain, H/E; original magnification, ×40. Scale bar = 200 μm.

Histopathological examination with hematoxylin and eosin or Safranin-O/fast green staining revealed that the cartilage in the ACLT + OVX, ACLT + OVX + 3 U calcitonin, and ACLT + OVX + 15 U calcitonin groups exhibited various degrees of pathological changes (Fig. 4). In the naïve (Fig. 4A) and 15 U calcitonin (Fig. 4B) groups, the cartilage appeared histologically normal. A thin, glistening, smooth lamina filled with flattened chondrocytes was observed, and no loss of proteoglycan was present in the matrix based on Safranin-O/fasting green staining. Specimens from the ACLT + OVX (Fig. 4C) groups exhibited significantly higher incidence rates and greater severity of surface erosions than those from the naïve group. The ACLT + OVX + 3 U calcitonin (Fig. 4D) and ACLT + OVX + 15 U calcitonin (Fig. 4E) groups showed marked reductions in the severity of cartilage lesions, but fibrillation and fissures extending into the superficial layer of cartilage were observed. The OARSI scores of the calcitonin-treated groups were significantly lower than in the ACLT + OVX groups (Table 1). In addition, calcitonin alone at 15 U showed no detrimental effects on the cartilage of naïve rats (Fig. 4B and Table 1). None of the experimental groups in the present study showed changes in the gross or histological appearance of the hip or ankle joints (n = 3) at 20 weeks after surgery (data not shown).

### Immunohistochemical detection of TGF-β1, confocal double-immunofluorescent staining of TGF-β1 and articular chondrocyte marker doublecortin in the articular cartilage

**Figure 5:**
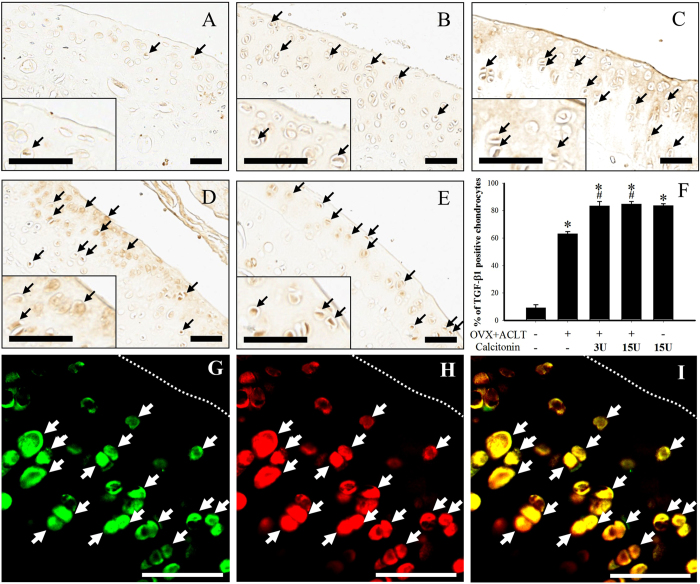
Distribution of TGF-β1 protein immunoreactivity and confocal double-immunofluorescent staining of TGF-β1 and articular chondrocyte marker doublecortin in the cartilage. Positive immunoreactivity of the TGF-β1 protein is indicated by the red-brown color (arrows). The panels show the distribution of anti-TGF-β1 immunoreactivity in the cartilage of the (**A**) naïve, (**B**) calcitonin 15 U, (**C**) ACLT + OVX, (**D**) ACLT + OVX + 3 U calcitonin, and (**E**) ACLT + OVX + 15 U calcitonin groups. All samples were stained with antibodies against TGF-β1 protein. (F) Quantitative analysis showed that calcitonin significantly enhanced the number of TGF-β1-positive cells in the cartilage of ACLT + OVX knees. Scale bar = 100 μm. *P < 0.05 compared with the naïve group, ^#^*P* < 0.05 compared with the ACLT + OVX group. Representative confocal immunofluorescence microscopy images showing the localization of TGF-β1 (G; green in color) and doublecortin (H; red in color) of articular cartilage in the ACLT + OVX + 15 U calcitonin group. Colocalization is indicated by yellow (I) and arrows. The confocal results showed that TGF-β1 was primarily co-localized with articular chondrocytes. Scale bars are 50 μm for all images.

The immunolocalization of phosphorylated TGF-β1 proteins in the cartilage specimens is shown in Fig. 5, and fewer TGF-β1-positive cells were observed in the naïve group (Fig. 5A) than in the ACLT + OVX group (Fig. 5B). In the ACLT + OVX, ACLT + OVX + 3 U calcitonin (Fig. 5C), ACLT + OVX + 15 U calcitonin (Fig. 5D), and 15 U calcitonin alone groups, 20 weeks after surgery, the numbers of TGF-β1-immunoreactive cells increased in the superficial and transitional cartilaginous zones. Calcitonin treatment increased ACLT + OVX-rats upregulation of TGF-β1 expression in cartilage chondrocytes (Fig. 5C,D). Quantification of the number of TGF-β1-immunoreactive cells revealed a significant increase in ACLT + OVX-induced upregulation of TGF-β1-positive cells in the calcitonin-treated ACLT + OVX and calcitonin alone groups (Fig. 5F, *P* < 0.05). To further confirm which cells were affected by calcitonin after ACLT + OVX, we used confocal microscopy to examine the chondrocytes in cartilage tissues. Localization of TGF-β1 (red color; Fig. 5G) and the chondrocyte marker protein, doublecortin (green color; Fig. 5H), in the articular cartilage of the ACLT + OVX + 15 U calcitonin group was determined using the double-labeling immunofluorescent staining method. The merged images of Fig. 5I indicated that TGF-β1 was colocalized with doublecortin (yellow; white arrow). The major cell type expressing TGF-β1 was chondrocytes in the articular cartilage tissues.

## Discussion

This is the first report showing that subcutaneous administration of calcitonin attenuated the development of OA and concomitant nociceptive behavior (secondary mechanical allodynia and weight-bearing distribution) and decreased inflammation in the knee joint in an experimental OA model in rats. Interestingly, calcitonin enhanced TGF-β1 expression in articular cartilage chondrocytes in this experimental model and in naïve rats.

The putative effect of calcitonin on the progression of OA has been investigated clinically, focusing on the positive effects on cartilage degradation^10^. Calcitonin was identified more than 40 years ago^25^, possesses potent anti-resorptive effects, and has been shown to be mediated by directly binding calcitonin to the calcitonin receptor on osteoclasts^26^. Calcitonin may directly target chondrocytes in the articular cartilage because human cartilage cells express the calcitonin receptor^27^. Signaling of calcitonin through the calcitonin receptor and binding to G-coupled receptors to activate adenylylcyclase can increase cAMP levels^28^, inhibit matrix metalloproteinase (MMP) activity^11^, and have chondroprotective potential^11^,^29^. Calcitonin also acts on osteoblasts to increase their proliferation and alkaline phosphatase activity *in vitro*^30^, which is associated with the increased synthesis and deposition of bone matrix collagen^30^. In *in vitro* culture of chondrocytes from human OA hips and knees, calcitonin appears to decrease collagenolytic activity and markedly stimulate the attachment of chondrocytes to fibronectin^31^. Badurski *et al*. were the first to report that salmon calcitonin reduced cartilage erosion and protected against cartilage glycosaminoglycan loss in experimental OA^32^. Manicourt *et al*. reported that the urinary level of deoxypyridinoline (a specific marker of bone resorption) significantly diminished in surgical-induced OA models by 3 weeks after subcutaneous injection with calcitonin^33^. Cartilage degradation may be assessed by measurements of the C-terminal telopeptide of type II collagen (CTX-II). Use of CTX-II as a marker for the progression of cartilage lesions and its direct relationship with radiological grades and clinical scores for OA have been demonstrated previously. Circulating high levels of CTX-II have been shown to be associated with OA and progression of the disease^34^. In *in vitro* studies of the culture of articular cartilage explants, direct chondroprotective effects were observed for calcitonin, by inhibiting matrix metalloproteinase activity and causing a dose-dependent decrease in CTX-II levels^35^ (ref). Studies by Karsdal *et al*. also demonstrated that oral salmon calcitonin given twice daily resulted in reductions in the markers of bone resorption and cartilage degradation (urine CTX-II)^36^. Thus, it is believed that early identification of CTX-II levels would be a useful tool for achieving early diagnosis of OA development and taking preventive action.

Various clinical and animal studies have reported different results for the effect of calcitonin on OA. Owing to the limited literature on calcitonin dosage, there is still no optimal calcitonin concentration, volume, or injection schedule that can reliably yield desired results. The subcutaneous injection was started 12 weeks after surgery because quantitative computed tomography (QCT)^37^ and dual x-ray absorptionmetry (DXA)^38^ have previously detected a reduction in bone mineral density in both femoral condyles and tibial plateaus at 12 weeks after ACLT, with a marked reduction in the internal compartment of the OA joint. Further studies are required to establish the optimal duration of calcitonin administration for OA. It is not sufficient to analyze the effects of calcitonin on only cartilage histopathology to determine its effectiveness on OA pathology. It is important to monitor changes in the subchondral bone because they are related to the changes in the cartilage. Joint unloading and early and persistent synovitis might cause a rapid loss of the subchondral trabecular bone. A 30% reduction in load bearing by an osteoarthritic knee has been shown to persist for at least 45 months after ACLT^39^. Calcitonin has been shown to improve the bone mineral density and volume fraction, as assessed by μCT in an ACLT dog model^40^. In the present study, the calcitonin dose used in rats was the equivalent dosage used in humans. Several animal models of OA are available, and each model mimics different mechanisms through which OA initiates and develops. There is an apparent need for an OA model that directly mimics a human form of the disease while simultaneously providing a convenient methodological tool for preclinical investigations. OVX interferes with estrogen production and increases bone and cartilage turnover. OVX alone has been shown to produce signs of OA in rats^4^ and to increase OA pathology in traumatic OA models in the rabbit^41^ and rats^42^. The prevalence of OA is higher in post-menopausal women than in men^43^. In the present study, establishment of the OVX + ACLT model resulted in joint instability, which could effectively mimic the pathological changes detected in human OA. Thus, this model is considered to be suitable for evaluating therapeutic agents for OA. The main limitation of the animal model used in our study is that we did not detect the pathology of osteoporosis but could only analyze the OA pathogenesis. In the present study, both the macroscopic and OARSI scores were significantly lower in the ACLT + OVX surgery plus calcitonin-treated animals compared to in those that received surgery alone. Subcutaneous calcitonin administration significantly reduced the severity of cartilage degradation in the ACLT + OVX knee. Our results indicate that calcitonin treatment had cartilage-protective effects in rat knee joints. Although previous studies have indicated that the effects of calcitonin include bone and both cartilage anti-catabolic and cartilage anabolic, the curative effects should be evaluated in long-term clinical trials. Several reports have implicated the involvement of the serotonergic^44^ and catecholaminergic systems^45^ in calcitonin-induced anti-nociception. In humans, the similarities between calcitonin and morphine-induced analgesia, as well as reports of calcitonin-induced elevation of plasma β-endorphine levels, suggest that the endogenous opiate system mediates the analgesic action of calcitonin^46^. In behavioral studies, repeated systemic injections of calcitonin inhibited formalin-induced hyperalgesia and OVX-induced hyperalgesia in rats^47^, while single injections had no effects^48^. Studies using rat and mouse models have indicated that OA pain is associated with the release of a sensory neuropeptide, calcitonin gene-related peptide (CGRP)^49^, and an increased CGRP release^50^, by joint afferents”. Bullock *et al*.^51^ suggested that the role of CGRP receptor systems might be an important target to modulate pain during OA^51^. Castro *et al*. used a quantitative method to associate joint pain with weight bearing in the ACLT rat model^52^. Our previous study showed that intra-articular injection of magnesium sulfate attenuated secondary mechanical allodynia and thermal hyperalgesia, in a rat model with collagenase-induced OA^23^. In the present study, ACLT + OVX caused a painful response in the injured right hind-paw in rats, causing the animal to redistribute its body weight in favor of the non-injured left limb. This nociceptive behavior was also characterized by decreases in the secondary mechanical allodynia threshold and significant decreases in weight distribution in the ACLT + OVX knee. Changes in joint width were measured to determine tissue swelling and as an index of inflammation^18^. The addition of calcitonin treatment reduced the knee joint width compared with the groups that received ACLT + OVX surgery alone. Since salmon calcitonin is a polypeptide, it may have some side effects on human body, such as decreased food appetite, nausea, and vomiting, among others. It may induce allergic response^53^. In the present study, we found that calcitonin may decrease nociceptive pain. The above data indicate that calcitonin decreases nociception and inflammation. However, the exact mechanism of the anti-nociceptive and anti-inflammatory activities of calcitonin requires further investigation. We apologize for the lack of comparison of the results for the contralateral left (non-injury) leg, including OA score, histopathological evaluation, and TGF-β level. In the original study design, there were 5 experimental groups, including the naïve, calcitonin 15 U, ACLT + OVX, ACLT + OVX + 3U calcitonin, and ACLT + OVX + 15U calcitonin groups. The results of OA score and the histopathological and immunohistochemical data of TGF-β were only compared amongst groups for the right (injury) legs. In our previous study^42^, the OA scores and histopathological findings of the contralateral non-injury legs showed normal findings.

The role of TGF-β in the pathogenesis of OA has gained attention in recent years. TGF-β1 has also been shown to down-regulate cartilage-degrading enzymes and counteract the catabolic cytokine interleukin (IL)-1, both *in vivo* and *in vitro*^54^. The ability of TGF-β to counteract IL-1 is also lost with age^55^. TGF-β1 generally enhances the chondrogenic differentiation of bone-marrow derived mesenchymal stem cells^56^. Therefore, TGF-β1 appears to be a good candidate for cartilage repair. The main TGF-β signaling route is through specific membrane receptors (activin receptor-like kinase receptors, ALKs) and its intracellular effectors, the Smad proteins^57^. Interruption of the TGF-β/Smad3 signaling pathway leads to chondrocyte hypertrophic differentiation and cartilage degeneration^14^. TGF-β induced nerve growth factor mRNA in bovine and human chondrocytes in an ALK5/Smad2/3-dependent manner^58^, and add a new layer to the complex pathogenic role of TGF-β in OA pathogenesis. TGF-β1 is considered essential for cartilage integrity and is present at high levels in normal cartilage but shows reduced expression in cartilage in OA^59^. Li *et al*. suggested that vitamin D supplementation showed protective effects in OVX-induced OA, partly through the TGF-β1 pathway^60^. Asporin inhibits TGF-β-mediated expression of cartilage matrix genes such as collagen type II and aggrecan; it also inhibits accumulation of proteoglycans^61^. Serra *et al*.^62^ demonstrated that overexpressing a dominant negative TGF-β receptor resulted in terminal chondrocyte differentiation and subsequent OA^62^. Taken together, these results elucidate the importance of endogenous TGF-β in maintaining cartilage integrity in OA. In the present study, we observed increase TGF-β1 expression in the calcitonin-treated ACLT + OVX groups, and treatment with calcitonin alone may increase TGF-β1 expression in naïve cartilage chondrocytes. This is the first demonstration that calcitonin treatment increases TGF-β1 expression in both ACLT/OVX-affected and naïve rat chondrocytes. Whether the TGF-β1 pathway is a crucial and multifaceted signaling pathway in OA pathogenesis requires further analysis. We hypothesize that TGF-β1 is at least partially responsible for OA development and nociception. These data, as well as the analgesic effects of calcitonin on the model, support that calcitonin is a new option for the treatment of OA.

## Additional Information

**How to cite this article**: Wen, Z.-H. *et al*. Calcitonin attenuates cartilage degeneration and nociception in an experimental rat model of osteoarthritis: role of TGF-β in chondrocytes. *Sci. Rep*. **6**, 28862; doi: 10.1038/srep28862 (2016).

## Figures and Tables

**Table 1:** Macroscopic and histological evaluation of articular cartilage of the femoral condyle and tibial plateau at 20 weeks after surgery.

Score Group	Macroscopic score	Osteoarthritis score (OARSI score)
ACLT + OVX (*n* = 8)	2.5 (1.4, 2.7)^*^	15.2 (7.6, 18.2)^*^
ACLT + OVX + 3U calcitonin (*n* = 6)	1.2 (0.4, 1.3)*^†^	5.6 (3.7, 7.2)*^†^
ACLT + OVX + 15U calcitonin (*n* = 7)	0.9 (0.5, 1.1)*^†^	4.4 (2.8, 6.1)*^†^
Naïve (*n* = 8)	0.5 (0.2, 0.6)	1.8 (1.2, 2.8)
Calcitonin 15U (*n* = 6)	0.6 (0.1, 0.4)	2.2 (1.5, 3.2)

Values are given as the mean ± SEM. The groups received surgeries and treatments as described in the Methods; **OARSI score:** Osteoarthritis Research Society International (OARSI) grading system. *P < 0.05 vs the naïve group; ^†^*P* < 0.05 vs the ACLT + OVX groups.
